# Inequalities in Uptake and Use of Digital Applications for Home-Monitoring of Neovascular Age-Related Macular Degeneration in an Elderly Visually Impaired Population: The MONARCH Study

**DOI:** 10.1167/tvst.13.3.2

**Published:** 2024-03-01

**Authors:** Ruth E. Hogg, Robin Wickens, Sean O'Connor, Eleanor Gidman, Elizabeth Ward, Tunde Peto, Benjamen J. L. Burton, Paul Knox, Andrew J. Lotery, Sobha Sivaprasad, Michael Donnelly, Chris A. Rogers, Barnaby C. Reeves

**Affiliations:** 1 Centre for Public Health, Queen's University Belfast, Belfast, UK; 2 Bristol Trials Centre, University of Bristol, Bristol, UK; 3 James Paget University Hospitals NHS Trust, London, UK; 4 University of Liverpool, Liverpool, UK; 5 Department of Clinical and Experimental Sciences, Faculty of Medicine, University of Southampton, Southampton, UK; 6 NIHR Moorfields Biomedical Research Centre, Moorfields Eye Hospital NHS Foundation Trust, London, UK; 7 Institute of Nursing and Health Research, Ulster University, Londonderry, UK; 8 Southampton Clinical Trials Unit, University of Southampton, Southampton, UK

**Keywords:** digital health, home monitoring, inequalities, inequity

## Abstract

**Purpose:**

To describe inequalities in the Monitoring for Neovascular Age-related Macular Degeneration Reactivation at Home (MONARCH) diagnostic test accuracy study for: recruitment; participants’ ability to self-test; and adherence to testing using digital applications during follow-up.

**Methods:**

Home-monitoring vision tests included two tests implemented as software applications (apps: MyVisionTrack and MultiBit) on an iPod Touch device. Patients were provided with all hardware required to participate (iPod and MIFI device) and trained to use the apps. Regression models estimated associations of age, sex, Index of Multiple Deprivation, strata of time since first diagnosis, and baseline visual acuity at study entry on outcomes of willingness to participate, ability to perform tests, and adherence to weekly testing.

**Results:**

A minority of patients who were approached were willing-in-principle to participate. Increasing age was associated with being unwilling-in-principle to participate. Patients from the most deprived areas had a 47% decrease in odds of being willing compared to those from the middle quintile deprived areas (odds ratio, 0.53; 95% confidence interval = 0.32, 0.88). Increasing age and worse deprivation were not consistently associated either with ability to self-monitor with the index tests, or adherence to weekly testing.

**Conclusions:**

Associations of increasing age and worse deprivation index were associated with unwillingness-in-principle to participate despite the provision of hardware’ highlighting the potential for inequality with interventions of the kind evaluated.

**Translational Relevance:**

The clear evidence of inequalities in participation should prompt future research on ways to encourage adoption of mobile health technologies by underserved populations.

## Introduction

The widespread adoption of digital health technologies is hoped to increase the efficiency of healthcare delivery. In Ophthalmology, providing regular, timely monitoring for patients receiving intravitreal injections of inhibitors of vascular endothelial growth factor for macular disorders, such as neovascular age-related macular degeneration (nAMD) and diabetic macular edema, has created considerable challenges. Such patients can require monitoring appointments for many years after treatment initiation. A hospital monitoring appointment typically involves an assessment of visual acuity (VA), retinal imaging including optical coherence tomography (OCT) and clinical examination (either in person or through OCT image review); this information enables the clinician to decide to either treat or continue monitoring. Self-monitoring with home-monitoring tests would offer the opportunity to provide a hospital appointment only when a trigger threshold for a test is reached. A variety of approaches have been considered including visual function tests implemented on tablets or smartphones and home OCTs.^1^^–^^4^

The MONARCH study (Monitoring for Neovascular Age-related Macular Degeneration Reactivation at Home) was a multicenter diagnostic test accuracy study that aimed to evaluate the effectiveness of a home-based monitoring system for patients with age-related macular degeneration (AMD).^5^ Tests evaluated included one paper-and-pencil test (KeepSight Journal [KSJ])^6^ and two tests implemented as software applications (apps: MyVisionTrack [mVT]^7^ and MultiBit [MBT])^8^ on an iPod Touch device (Apple, Cupertino, CA, USA). The device, equipped with internet access via a MiFi device, allowed the data to be automatically transmitted to a server and then to the study database. Although the primary objective of the study was the diagnostic accuracy of the chosen tests, we were aware that attempting to implement digital technologies in this cohort had the potential to create inequality. In particular, digital exclusion and low socioeconomic status may exacerbate existing inequalities^9^^–^^11^ because digital access and skill are considered foundational social determinants of health.^12^ During the study design phase, the small percentage of regular internet and smartphone users in the UK at that time, in particular, was considered a potential threat to the study.^13^ We were especially concerned that potential participants might feel alienated by the technology and would not be prepared to try out the solutions we proposed. So from the outset, we sought to determine the extent to which the technology was a barrier to consent and participation to enable those in the future who sought to implement such technologies to mitigate such barriers.

Therefore, as a secondary objective, the MONARCH study explored whether inequalities (by age, sex, social economic status, and VA) existed in recruitment to the study and impacted the ability of participants to do the app-based tests during follow-up or the adherence of participants to weekly testing.

## Methods

The full protocol,^5^ main results (RE Hogg, manuscript submitted, 2024), overview of challenges in implementing home monitoring (BC Reeves et al., manuscript submitted, 2024) and qualitative findings^14^ are published elsewhere. The study was conducted at six NHS Hospitals in the United Kingdom. Ethical approval was granted by the Northern Ireland Health and Social Care Research Ethics Committee A (reference number: 17/NI/0235) on January 29, 2018, and the study adhered to the Declaration of Helsinki.

We designed the study to include the following features to try to minimize the extent to which familiarity with technology could be a barrier to home monitoring:

a) We included a simple paper-based home monitoring test (KSJ), which we hoped would feel familiar to participants. This test involved a series of puzzles that required participants to use their near vision correction.
b) We provided an iPod Touch device preloaded with two apps (mVT^7^ and MBT)^8^
c) We also provided a mobile broadband device so that participation was not limited by the lack of home Wi-Fi. The device had a simple on/off switch; the only things that a participant needed to remember to do was to keep the device charged (a main micro-USB charger was provided) and to switch on the device before performing the home-monitoring tests that use the iPod. The iPod interacted with the mobile broadband device automatically to transmit data.
d) We explained the use of the devices during an initial training and information session with each potential participant, provided a helpline for participants to call in the event of experiencing difficulty, and also provided an option for additional training if requested.

### Methods Relevant to the Exploration of Inequalities

#### Patient Identification

Potential study participants were identified by local clinical research teams from established clinical databases of patients and by reviewing lists for outpatient clinics. Potential participants were screened for eligibility^5^ by the healthcare team through review of their medical notes and any existing retinal imaging.

Potential participants were sent by post or given an invitation letter and patient information leaflet describing the study. An appropriately trained and qualified member of the local research team (e.g., study clinician/research nurse/optometrist) discussed the study with them by telephone or in person. We had ethics approval to collect a minimum dataset for all potential participants who were provided with a patient information leaflet. Together with a unique study number, the dataset comprised reason(s) for non-participation (e.g., reason for being ineligible or patient refusal) and equality monitoring data (age, sex, ethnicity, index of multiple deprivation and most recent VA for each eye) but no identifiable information.

#### Training and Equipment

Verbal consent to attend the further information and training session was taken by a member of the local research team and recorded in the patient's hospital record. The information and training session was led by an appropriately qualified member of the local research team with experience of working with patients. At the information and training session, the potential participant was shown the equipment and how it should be used for the study and asked to self-monitor weekly with each of the three tests. After obtaining written informed consent, the participant was provided with the following to take home: the iPod touch, a lens cloth, an eye patch, stylus pen, the KSJ, and the mobile Wi-Fi broadband router. The Apple iPod was preloaded with two apps, Multibit (MTB; a near acuity threshold test of neuro-retinal damage) and MyVisionTrack (mVT; a shape discrimination test that measures hyperacuity).^5^

Patients were followed up for at least six months. Participants continued to have usual care (i.e., review of disease activity and treatment if required) in NHS monitoring clinics. Retinal imaging was also carried out as required to inform usual care management decisions. Local site teams collected data for study and fellow eyes at each usual care follow-up visit. A management decision was a decision about the status of an nAMD lesion, the treatment plan, or both.

#### Study Delivery

Participants were contacted before the management decision for each follow-up visit was made. Participants were telephoned before (maximum of five working days before retinal imaging) or seen in clinic before having an appointment. A member of the local research team asked the participant questions on how they felt their vision had been since their last visit, whether the participant had been carrying out home monitoring, whether the participant had experienced any problem with home monitoring, to confirm the participant's willingness to continue and whether there was need for retraining.

The following outcomes were investigated as measures of uptake of home monitoring tests:

(i) *Inequalities in recruitment.* Ethical approval included permission for the collection of age (under 70 years, 70 to 79 years, and 80 years and older), sex, and index of multiple deprivation (IMD) rank for all screened patients to explore potential inequalities. The outcome of “willingness-in-principle to participate” was defined as an approached patient agreeing to attend a research visit for training.
(ii) *Inequalities in ability to do tests during follow-up.* The outcome of the ability to perform an index test was defined as the proportion of monitoring visits for which some valid index test data were available.
(iii) *Inequalities in adherence to weekly testing.* The outcome of adherence was calculated as the number of weeks with a valid home monitoring test over the total number of weeks between the preceding management visit and the most recent management visit.

Inequalities in the ability to do the tests and adherence to weekly testing were investigated for each test separately and only among study participants.

The following potential predictors of the above outcomes were also collected during the study from the hospital records and entered into the study database:

• Patient sex: as recorded on the database
• Patient age: calculated as age at consent
• Exposure to technology: use at least weekly of any of: smart-phone, internet; only collected for study participants so not investigated as an predictor of recruitment.
• Stratum of time since initiation of anti-vascular endothelial growth factor treatment: six to17 months; 18 to 29 months; 30 to 41 months.
• VA at diagnosis: VA at diagnosis was calculated into three categories, based on the worst eye in the study for the patient. The categories were: Snellen better than or equal to 6/18; worse than 6/18 and better than 6/24; and worse than or equal to 6/24.

### Statistical Analysis

Regression models at the level of the patient explored the influences of age, sex, IMD, stratum of time since first diagnosis, and baseline VA at diagnosis on the outcomes described above. Associations of these predictors with willingness-in-principle to participate in screening (when first approached) was analyzed by logistic regression. The ability of a participant to complete a test and adherence to weekly testing were proportions, which were analyzed using a fractional logit approach using a general linear model with a logit link and binomial distribution with the binomial denominator included. The ability and adherence models were performed for each test separately. The influence of all factors are reported as odds ratios (ORs) with 95% confidence intervals (CIs). (The fractional logit models provide odds ratios when the coefficients are exponentiated. This approach is more suitable than linear regression when outcomes are bounded by 0 and 1.)

The IMD was used as an indicator of the participant's socio-economic status. However, IMD ranks cannot be directly compared between UK countries. To allow comparison of Belfast Northern Ireland with English IMD ranks, adjusted IMD ranks were used, by normalizing 2010 NI IMD data to the 2015 English IMD. The approach required back-converting available (English) IMD ranks on the MONARCH database to Lower Layer Super Output Areas geographies, allowing linkage to an adjusted IMD data source (https://data.bris.ac.uk/data/dataset/1ef3q32gybk001v77c1ifmty7x ). Because the exposure to technology questions were only asked after consent, the indicator could not be examined in the analysis of inequalities on willingness-in-principle to participate.

## Results

### Inequalities in Recruitment

We recruited 297 (31.5%) participants from 943 potential participants who were approached and eligible based on data available at screening. During mapping to IMD, seven patients were identified as having erroneous IMD ranks: four because of residing in the Isle of Man and three because of having out-of-range IMD ranks. These patients were excluded from the analysis. The characteristics of the remaining 936 patients are described in Table 1. Of the 936 patients with complete data, 291 (31.1%) were willing-in-principle to take part.

**Table 1.** Demographic Characteristics of Participating Patients Versus Nonparticipating Patients Eligible at Screening

	Overall (n = 936)	
	Not Participating	Participating
n	645 (69%)	291 (31%)
Gender		
Male	220 (34%)	119 (36%)
Female	425 (66%)	172 (59%)
Age		
Mean (SD)	78.1 (7.5)	74.9 (6.6)
Minimum age	50	54
Maximum age	97	93
Ethnicity		
White/Caucasian	359 (56%)	201 (69%)
Asian/Asian British	1 (0%)	—
Mixed/Multiple ethnic groups	8 (1%)	3 (1%)
Data not available	277 (43%)	87 (30%)
Snellen Visual Acuity		
Snellen ≥ 6/18	532 (82%)	258 (89%)
Snellen < 6/18 and > 6/24	61 (9%)	19 (7%)
Snellen ≤ 6/24	52 (8%)	14 (5%)

Percentages given are across all patients assessed as eligible at screening and should be referred to the denominators at the top of each column.

For patients with two potential study eyes, the better seeing eye is used.

Because of the small number of participants under 60 years of age (11 participants; no participation = 6, participation = 5) and over 89 years of age (32 participants; no participation = 30, participation = 2), participant age was split into three categories for the analysis: under 70 years, 70 to 79 years, and 80 years and older.

**Figure 1.**
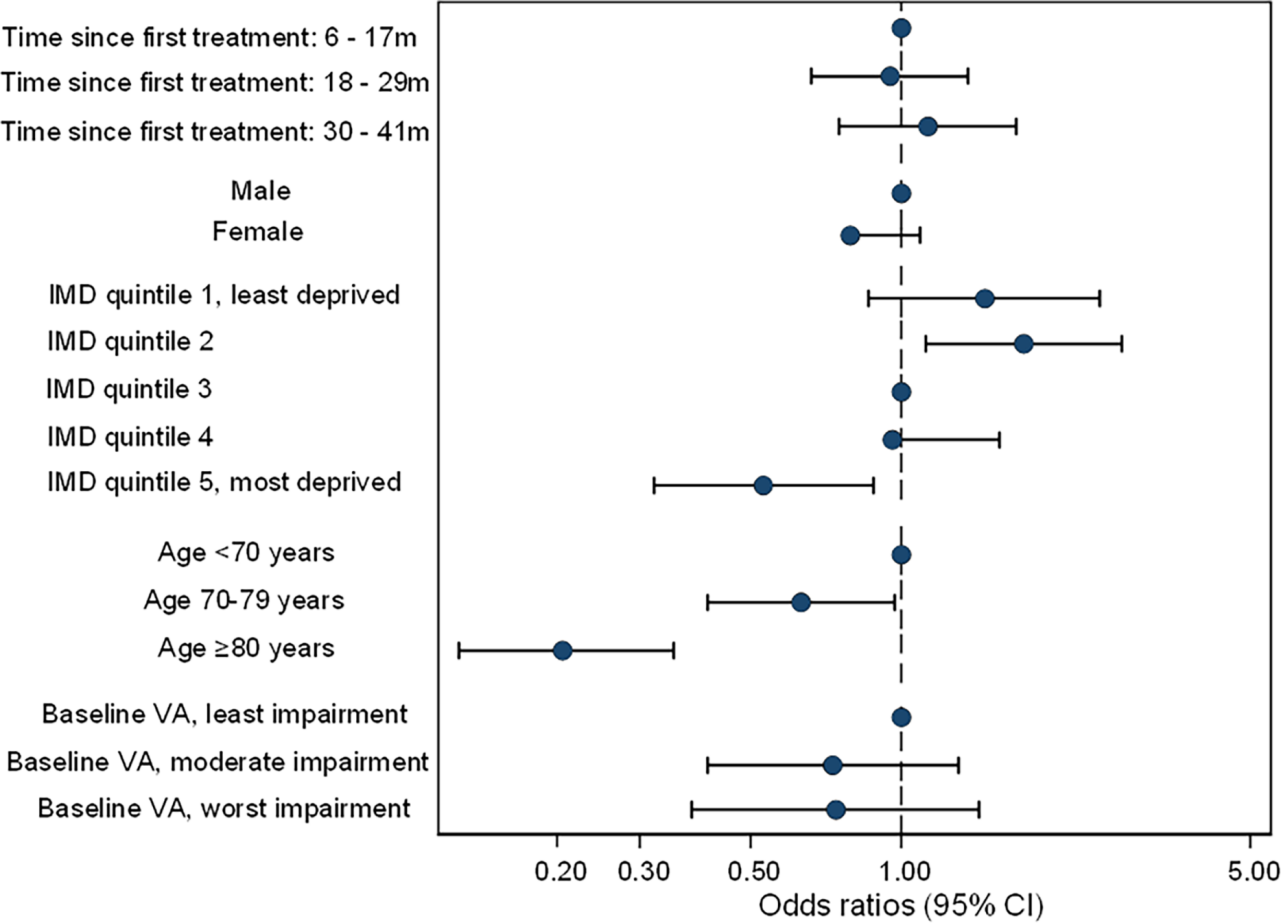
Impact of inequalities on participation.

Associations of predictors of interest with willingness-in-principle to participate are shown in Figure 1. Age was a significant predictor of willingness to participate (overall of the effect of age category, χ^2^ = 50.5 p < 0.001). Patients aged 80 years or older were seen to have significantly decreased odds of being willing compared to patients < 70 years old (OR = 0.21; 95% CI, 0.13, 0.35; *P* < 0.001). Patients between 70 and 79 years of age had 37% decreased odds (OR = 0.63; 95% CI, 0.41, 0.97; *P* = 0.037).

There was no significant effect of time since the first treatment for nAMD on the odds of willingness to participate in the study. There was a 21% decrease in the odds of women participating in the study, although this was not significant (OR = 0.79; 95% CI, 0.58, 1.09; *P* = 0.149). The overall effect of IMD quintile was highly significant (χ^2^ = 24.3, *P* < 0.001). Patients from the most deprived areas (IMD quintile 5) had a 47% decrease in odds of being willing compared to those from the middle quintile deprived areas (OR = 0.53; 95% CI, 0.32, 0.88); those in the second quintile had a 1.8-fold increase in odds of participation (OR = 1.76; 95% CI, 1.12, 2.76).

Decreased odds of participation were seen when potential study eyes had VA worse than 6/18 at baseline compared to better than or equal to 6/18. However, this impact was not significant overall (χ^2^ = 1.90, *P* = 0.387).

### Frequency of Testing and Inequalities in the Ability To Test During Follow-Up

Participants were asked about their use of everyday items of digital technology. Most participants used a smartphone, tablet, laptop/home computer, internet, e-mail, or social media at least weekly (266/297 [89.6%]) (BC Reeves et al., manuscript submitted, 2024).

**Figure 2.**
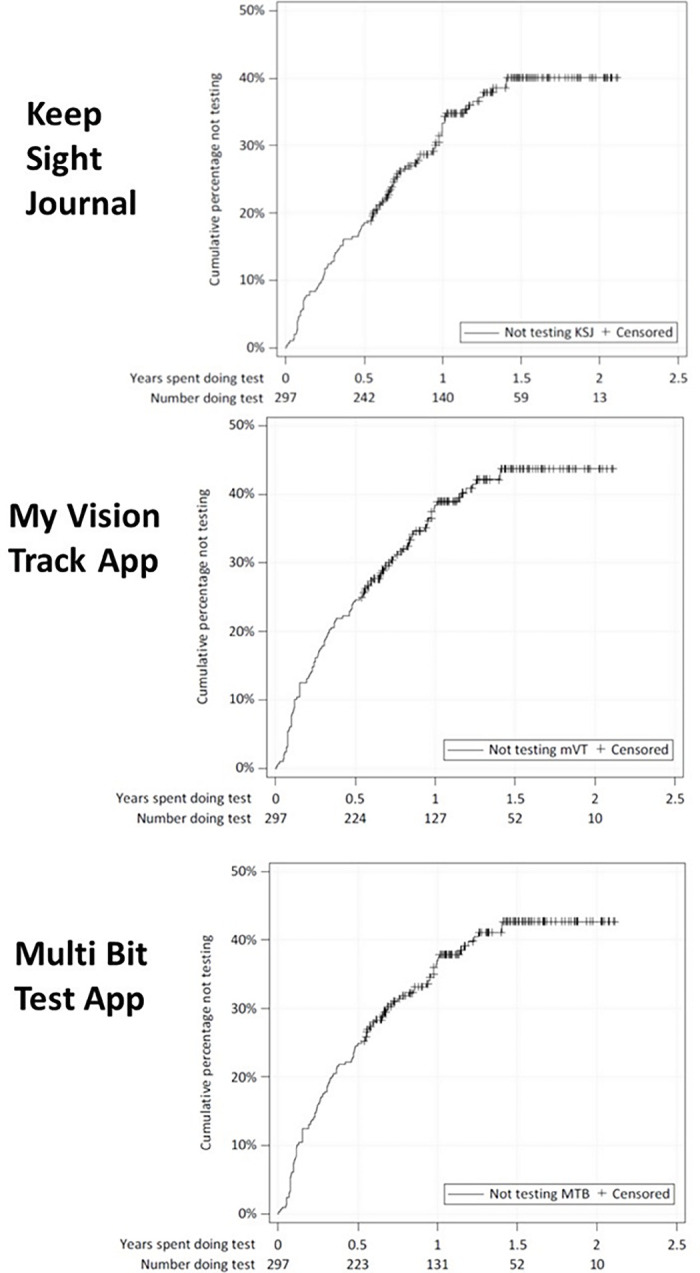
Time to stopping home monitoring with the Keep Sight Journal, My Vision Track and Multi Bit Test.

Frequencies of testing and test completion for the three home monitoring tests are shown in Table 2. The median interval between completing the KSJ was 7 days (inter-quartile range [IQR], 7, 7). The median testing frequency for the apps was four times per month (IQR, 1, 4). Test data from the apps continued to be transmitted for about 60% of study eyes by 18 to 24 months after starting to test; 56.2% and 59.3% of expected weekly tests were completed for MBT and mVT, respectively. Figure 2 shows the time to stopping testing for each of the tests.

**Table 2.** Weeks When Participants Were in the Study, Weeks When Test Data Were Available, and Frequency of Testing

Home-Monitoring Test	Total Patient Weeks in Study	Weeks With Complete Data for ≥1 Test	Weeks With Some Data for ≥1 Test^*^	Test Frequency, Median (IQR)
Keep Sight Journal	15,624	9341 (59.8%)	1355 (8.7%)	7 (7, 7)^†^
Multibit app	18,937	10,648 (56.2%)^‡^	—	4 (1, 4)^§^
My Vision Track app	18,937	11,236 (59.3%)^‡^	—	4 (1, 4)^║^

* There were several elements to a weekly KSJ entry and entries for some weeks were incomplete, rather than not done at all.

^†^Test frequency is described as the median number of days (and IQR) between KSJ entries.

‡ Participants sometimes tested with the app more than once per week; the study captured data for a total of 16,672 Multibit test occasions.

§ Participants sometimes tested with the app more than once per week; the study captured data for a total of 17,482 My Vision Track test occasions.

║ Test frequency is described as the median number of tests (and IQR) per month between KSJ entries.

The results of the analysis of participant ability to perform the app tests are shown in Table 3. VA stratum at baseline could not be included for the MTB because of it being perfectly predictive for the worse than or equal to 6/24 VA stratum, and it was not significantly associated with ability to test with the mVT. There were no associations of ability to test with time since first treatment for nAMD, sex, age, IMD, or exposure to technology on participant ability to perform home testing was seen for any test.

**Table 3.** Impact of Inequalities on Participants’ Ability to Perform the MTB and Mvt Tests (n = 297)

	Multibit			My Vision Track		
Predictor	Odds Ratio	95% CI	*P* Value	Odds Ratio	95% CI	*P* Value
Time since first treatment for nAMD						
6–17 months	1.00			1.00		
18–29 months	0.66	(0.31, 1.41)	0.284	0.73	(0.35, 1.53)	0.410
30–41 months	1.38	(0.60, 3.15)	0.449	0.99	(0.42, 2.33)	0.974
Sex						
Male	1.00			1.00		
Female	0.67	(0.33, 1.39)	0.285	0.90	(0.44, 1.81)	0.762
IMD Quintile						
1 (least deprived)	2.05	(0.75, 5.59)	0.162	2.30	(0.81, 6.55)	0.120
2	1.37	(0.52, 3.64)	0.522	0.90	(0.36, 2.27)	0.825
3	1.00			1.00		
4	1.08	(0.45, 2.61)	0.865	0.86	(0.36, 2.06)	0.732
5 (most deprived)	0.66	(0.23, 1.91)	0.438	0.91	(0.30, 2.70)	0.860
Age						
<70	1.00			1.00		
70–79	0.95	(0.36, 2.51)	0.922	1.29	(0.51, 3.24)	0.586
80+	0.61	(0.20, 1.82)	0.371	0.86	(0.31, 2.41)	0.778
Baseline VA Strata						
Better than or equal to 6/18	—	—	—	1.00		
Worse than 6/18 & better than 6/24	—	—	—	0.54	(0.20, 1.44)	0.219
Worse than or equal to 6/24	—	—	—	5.84	(0.70, 48.7)	0.103
Exposure to technology						
No exposure						
Exposure	2.05	(0.69, 6.07)	0.194	1.62	(0.60, 4.36)	0.336

### Inequalities in Adherence to Weekly Testing

For adherence to testing with the apps (Table 4), there were overall associations with VA stratum at baseline for the MTB and mVT (χ^2^ = 20.9 and 11.3, *P* < 0.001 and 0.004, respectively, with adherence for the worse than or equal to 6/24 VA stratum being significantly lower than better than or equal to 6/18 VA stratum. There were no associations of adherence to testing with time since first treatment for nAMD, sex, age, or exposure to technology.

**Table 4.** Impact of Inequalities on Participants’ Adherence to Weekly Testing With the Multibit and My Vision Track Tests (n = 297)

	Multibit			My Vision Track		
Predictor	Odds Ratio	95% CI	*P* Value	Odds Ratio	95% CI	*P* Value
Time since first treatment for nAMD						
6–17 months	1.00			1.00		
18–29 months	1.13	(0.70, 1.83)	0.615	1.16	(0.73, 1.86)	0.521
30–41 months	1.09	(0.64, 1.88)	0.746	1.04	(0.60, 1.80)	0.890
Sex						
Male	1.00			1.00		
Female	0.90	(0.58, 1.38)	0.620	1.03	(0.68, 1.58)	0.883
IMD Quintile						
1 (least deprived)	1.18	(0.60, 2.33)	0.627	1.32	(0.69, 2.54)	0.403
2	0.95	(0.52, 1.73)	0.857	0.87	(0.47, 1.60)	0.653
3	1.00			1.00		
4	0.69	(0.37, 1.29)	0.245	0.68	(0.36, 1.29)	0.240
5 (most deprived)	0.98	(0.46, 2.08)	0.949	1.15	(0.56, 2.38)	0.699
Age						
<70	1.00			1.00		
70–79	0.97	(0.58, 1.64)	0.917	1.05	(0.62, 1.77)	0.850
80+	0.85	(0.47, 1.55)	0.593	1.12	(0.62, 2.03)	0.715
Baseline VA Strata						
Better than or equal to 6/18	1.00			1.00		
Worse than 6/18 & better than 6/24	1.12	(0.55, 2.28)	0.757	1.09	(0.51, 2.31)	0.824
Worse than or equal to 6/24	4.14	(2.25, 7.62)	<0.001	3.10	(1.60, 6.01)	0.001
Exposure to technology						
No exposure	1.00			1.00		
Exposure	1.66	(0.81, 3.37)	0.164	1.77	(0.91, 3.44)	0.093

## Discussion

In the MONARCH study, we found that a minority of patients who were approached were willing-in-principle to participate. Increasing age and deprivation index for home address were associated with being unwilling-in-principle to participate. However, IMD quintile and age were not consistently associated with either ability to self-monitor with the app tests, or adherence to weekly testing. Participants with the worst VA at baseline were less adherent, possibly due to being closer to threshold and finding the experience of testing more difficult and dispiriting, making them less likely to engage or persevere throughout follow-up.

The COVID-19 pandemic significantly hastened the adoption of telemedicine and digital health interventions alongside heightened concern about the impact of the digital divide and its potential to increase health inequalities.^11^^,^^15^ A recent study conducted at Moorfields Eye Hospital, which also used the mVT test, explored factors that were associated with engagement.^16^ The app was offered to consecutive patients who possessed a tablet or smartphone, of the 417 patients given the app and told to test twice weekly, 258 (61.9%) registered to use the app and tested at least once (“active” users) with just 166 testing at least twice weekly for a continuous period of at least four weeks (“active” and “compliant” users). Among patients who were active users, engagement was assessed as either compliance (a continuous period of at least four weeks in which tests were performed twice weekly) or use rate (total number of tests conducted by a patient divided by the overall period in weeks since the app was prescribed). They found that engagement was positively associated with high comfort with technology, white British ethnicity, visual acuity, neovascular age-related macular degeneration diagnosis, and the number of intravitreal injections whereas engagement was negatively associated with increased age. Given the pragmatic nature of this study introduced as a service quality improvement during COVID 19 in May 2020, it is difficult to directly compare the results with our study, although the relationship with visual acuity and adherence is similar. Their findings with respect to race, age, and digital literacy do raise concerns about these types of interventions introducing inequity if adopted as usual care by a health service.

We provided both the hardware for accessing the apps (iPod touch) and internet access (MiFi device and network contract) to mitigate potential inequalities in participation in our study. Despite this, those who chose to participate had a high prevalence of digital literacy and internet access, so providing these resources alone was insufficient to engage those without such experience (BC Reeves et al., manuscript submitted, 2024). This has significant implications as publications focused on mitigating digital inequality often suggest the provision of a loaner table with data plan remuneration as a solution to enhancing equity and inclusiveness in clinical care and research.^17^ Our study provides a cautionary warning that there is unlikely to be any such quick fix to mitigating digital inequality. A recent scoping review of inequities in health care services caused by the adoption of digital health technologies,^18^ identified two major dimensions, (i) access to and availability of digital health technologies by different social groups and (ii) health outcomes caused by lack or limited access to digital health technologies, highlighting the multifactorial nature of the problem. Countermeasures to lessen health inequities because of the introduction of digital interventions therefore need to be multilevel such as ensuring that government agencies and medical institutions provide resources such as hardware and internet connectivity and ensuring that disadvantaged groups are consulted during the design and implementation stage to ensure that the design maximizes acceptability. They also suggest programs to target eHealth literacy through the provision of relevant technical tools and volunteers to develop participants' self-confidence and skills. Interestingly, the authors highlighted the importance of public libraries as public spaces because they provide digital access, health information resources, as well as services and staff that may be able to assist with difficulties.

Scanzera et al.,^19^ based in Chicago highlighted the issue of the digital divide magnifying inequalities during the COVID-19 pandemic and the adoption of teleophthalmology, issues identified including smartphone ownership, availability of home broadband, and lack of digital literacy, meaning that Black and Latinx participants were more likely to have problems accessing virtual at-home visits. The presence of visual impairment made it additionally difficult because of the inability to read the font displayed on the screens or open specific applications. Simple solutions do not exist to mitigate inequities, and a multisystem and multilevel approach is required. Richardson et al.^20^ have recently presented a comprehensive framework for digital health equity, examining key digital determinants of health at the individual, interpersonal, community, and societal levels and embedding this within a leading health disparities framework. They hope that it provides a tool to enable digital healthcare leaders in industry, academia, policy, and the community to develop interventions that reduce inequity rather than increase it. They also provide a case study of the framework applied for remote patient monitoring providing solutions at an individual, interpersonal, community, and societal level.

### Strengths of this Study

We attempted to minimize inequality by providing a device for self-monitoring compared to most other studies of home monitoring to date, which required participants to use their own devices. We attempted to avoid access to the internet being a barrier to participation by providing an additional MiFi device. Assessing the impact of inequalities was integrated into the study design and enabled the collection of the impact of inequalities on willingness to participate, which is rarely reported on in these contexts.

### Weaknesses of This Study

Although this was a multicenter study recruiting participants from hospitals in both England and Northern Ireland, there was insufficient racial diversity to study the influence of this factor on the evaluated outcomes evaluated; more than 50% of participants were recruited by two of the sites with White-dominated catchment populations. However, of those deemed ineligible at screening, none were because of an English language barrier so even in the English Hospitals where racial diversity is prevalent, this factor did not seem to feature in willingness-in-principle to participate. There were more females than males in the overall study, in keeping with the higher prevalence of AMD in females together with longer life expectancy. We also recognize that although we assumed that those who were unwilling to participate in the study did so mainly because of a reluctance to engage with the digital technology, it is well documented that minoritized populations are more hesitant to take part in research more generally.^21^

## Conclusions

It is likely that in future studies to address potential inequities, it will be important to ensure that remote monitoring devices are accessible to all older patients, regardless of their socioeconomic status, health literacy, language, or cultural background. However, addressing these challenges would require a multifaceted approach, including appropriate education and training, device design according to best practice and accessibility and provision of appropriate support services such as a helpline. This would require healthcare providers, caregivers, and technology companies to work together to ensure that older patients can benefit from the advantages of remote monitoring technologies while overcoming these challenges.
